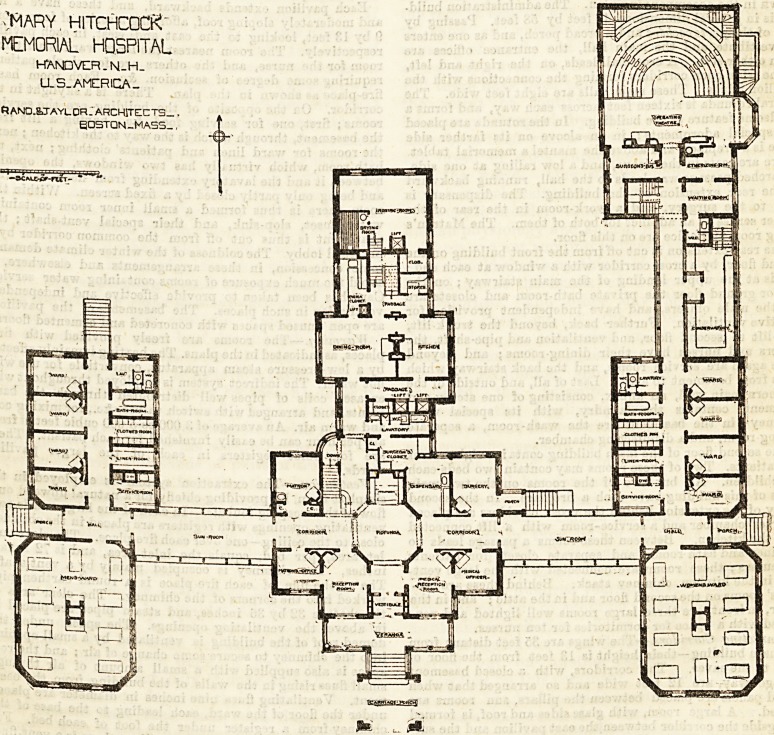# The Mary Hitchcock Memorial Hospital, Hanover, N.H., U.S.A.

**Published:** 1892-04-02

**Authors:** 


### HOSPITAL CONSTRUCTION.

### THE MARY HITCHCOCK MEMORIAL HOSPITAL, HANOVER, N.H., U.S.A.

This hospital is designed by its founder, Mr. Hiram Hitchcock, of New York, also a resident of Hanover, and Trustee of Dartmouth College, as a memorial to his deceased wife. It is to be an adjunct to the Medical School of the College, to which it is a munificent benefaction, as well as to the town and village in which it is placed.

The hospital consists of four distinct buildings, a central, or administration block, with an ell of two stories and an attic; two one-story pavilions connected with the central building by open corridors, and a surgical building designed for the special purposes of the Medical College. The capacity of the hospital is 36 beds.

All the exterior walls are hollow, and the plastering is applied directly to the interior surface of the bricks and tiling, with a finish of "adamant," affording a fine, hard, non-absorptive surface, like cement, that does not check or crack. The basement and foundation walls are of granite of the locality.

This system of construction avoids the use of heavy iron beams, and brick or terra-cotta arches of great weight, and renders the whole structure substantially a fire-proof monolith. It has been recently introduced in America by Mr. Guastavino, an educated Spanish architect, as an adaptation to modern use of the early Italian and Spanish dome-construction. It has been used in part in a number of important buildings, but this is the first one, and the first hospital, in America which has been planned especially for the use of this method throughout.

The architects, Messrs. Rand and Taylor, of Boston, have done previous excellent work in the designing of hospitals and asylums. The cost of the three buildings constituting the hospital proper will approximate ninety thousand dollars (90,000 dols.), and about twenty thousand dollars (20,000 dols.) more for the surgical annex. Deducting the extra cost of the special fire-proof construction about seventy-five thousand dollars (75,000 dols.) would remain, approximately, as a basis of comparison with the cost of a "slow burning" building of like capacity as commonly built. The administration building particularly has a special memorial character, and is liberally designed; and the materials have been generously chosen for the whole structure. The plan provides for the convenient enlargement of the hospital by additional buildings and extension of corridors.

*Site and Aspect.*—The buildings are situated upon a large open area, including over one hundred acres of land, of which all that is desirable is devoted to the hospital. The aspect is nearly south, with streets at a generous distance on the front and west, and the ground slopes downward from near the building to the east and north. Beyond the street to the west it descends irregularly to the banks of the Connecticut River one-sixth of a mile away; and there are pleasing views of more distant hills and mountains in this and other directions.

*Subsoil, Drainage, and Water Supply.*—The foundations of the structure rest upon the top of a bed of clay under a thin surface layer of sandy loam; next to the group of buildings, on the front and sides, the surface grade is raised to within four feet of the first or ground floor, and slopes gently to the natural level of the surrounding grounds. In the rear, and in the spaces there between the buildings, the surface is near the level of the basement floors. The natural drainage is excellent, and the main sewer leads through a settling tank to a filtration tank near the river at a distance of one-half of a mile. The sewer is of earthenware drain tiles, and at a distance from the buildings a flushing tank is placed. The water supply is at present furnished in small part by an aqueduct for drinking purposes and the like ;and by collecting the rain water from the roofs in cisterns for common uses.

*General Arrangement.*—The general arrangement of the buildings, with detail of the ground floor throughout, is shown in the accompanying plan. The administration building is in general dimensions 42 feet by 58 feet. Passing by way of the *porte cochère* and a broad porch, and as one enters the vestibute and the main hall, the entrance offices are upon either side. A cross hall leads, on the right and left, to the wide open corridors forming the connections with the pavilion wings. These main halls are eight feet wide. The central rotunda is sixteen feet across each way, and forms a handsome feature of the building. In the rotunda are placed the special adornments; in an alcove on its farther side there is a fireplace, and above the mantel a memorial tablet. There are seats in the alcove, and a low railing at one side. An arched passage-way leads to the hall, running backward to the rear extension of the building. The dispensary is next to the surgery, and a work-room in the rear of the former serves as an adjunct for both of them. The Matron's living room and office are on this floor.

The rear extension is cut off from the front building on the second floor, by a cross corridor with a window at each end; this is at the upper landing of the main stairway; on the first or ground floor the private bath-room and closets are for the male officers, and have independent provision for effective ventilation. Further back, beyond the trunk-lift, food-lift to second floor, and ventilation and pipe-shaft, the officers and nurses have their dining-rooms; and beyond these again are service rooms, and the back stairway which leads from basement to attic. Last of all, and outside of the northern main wall, an annex, consisting of one storey and basement, contains the laundry, with its special vent-chimney; in the basement are the wash-room, a separate rinsing-room, and a disinfecting chamber.

The second floor of the main building contains eight rooms for patients. Two of these rooms may contain two beds each for children. All but two of the rooms on the two main floors of this building have each a fireplace. In the second storey of the extension behind the cross corridors are placed a nurses' chamber and a service-room with a lift connected with the kitchen. Between these rooms a passage leads to the linen and bath-rooms, and separate closets for patients and nurses; these rooms are connected with the large vent-flues in the kitchen chimney stack. Behind these are servants' rooms on the second floor and in the attic; and in the latter, in front, are three large rooms well lighted and ventilated with a space for dormitories for ten nurses.

*Connecting Corridors.*—The wings are 35 feet distant from the main building—their height is 13 feet from the floor of ward to the eaves. The corridors, with a closed basement passage way, are 12 feet wide and so arranged that when glazed panels are placed between the pillars, sun rooms are formed. A large room, with glass sides and roof, is formed alongside the corridor between the east pavilion and the surgical building. This will serve as a sun-room, or conservatory, as may be designed.

*Pavilions.*—The principal sick wards are in the pavilions. The unique shape of the wards is designed to combine the merits of the round and rectangular form. They have the advantage of the latter in the arrangement of furniture, &c.; and the cutting off of the angels at the four corners, with the arching of the ceilings gives the practical effect of the round ward. The small size of the wards, each containing ten beds, aids in gaining its effect. Each pavilion ward is so placed that all of its walls are exposed to light and air except a few feet at one corner. The southern aspect permits the entrance of the sunlight all day in each ward alike.

The principal ward is 28 by 36 feet in the clear; this allows an average of 100 square feet of floor araa for each of the ten beds. The height of the ceiling is 11 feet at the sides of the room, rising by a short curve to near its maximum height, which is 14 feet at the centre, where it is pierced by the central chimney. This allows about 1,300 cubic feet of air space per bed. The floors of this building are of hard pine, except in the lavatory and water-closet, where tiles or marble mosaic is used, and the wood finish is simple and of varnished cypress. All corners are rounded, avoiding grooves for the lodgment of dust. All the corners of the rooms are rounded also, including the joining of the baseboards with the floor, and the corners of the chimney in the large ward.

The windows are 3 by 7¾ feet in the clear, giving an average of 19⅜ square feet per bed, of effective glazed surface. There are double runs of sash in the windows of all wards and rooms for patients and all living rooms, as the climate is cold in winter, when a temperature of 25 deg. Fah. is not infrequent.

Each pavilion extends backward, and these have a low and moderately sloping roof, affording a series of four rooms 9 by 13 feet, looking to the east and west, in each pavilion respectively. The room nearest the large ward is a sitting-room for the nurse, and the others are for single patients requiring some degree of seclusion, &c. Each room has a fire-place as shown in the plan. There is a skylight in the corridor. On the opposite of the building are the service-rooms; first, one for serving food, &c., having a lift from the basement, through which is the way to the kitchen; next, the rooms for ward linen and patients' clothing; next, the bath-room, which virtually has two windows, the opening between it and the lavatory extending from floor to ceiling, and being only partly closed by a fixed screen. Within this room there is thus formed a small inner room containing water-closet, slop-sink, and their special vent-shaft; this apartment is thus cut off from the common corridor by a practical lobby. The coldness of the winter climate demands some concession, in these arrangements and elsewhere, to avoid too much exposure of rooms containing water service. Care has been taken to provide effective and independent ventilation in such places. The basements of the pavilions are open unused spaces with concreted and cemented floors.

*Warming.*—The rooms are freely provided with fire-places, as indicated in the plans. The heating is mainly effected by a low-pressure steam apparatus, convertible for use with hot water. The indirect system is employed throughout with encased coils of pipes well distributed through the basements, and arranged with switch valves, &c., for mixing cold and warm air. An average of 3,000 to 5,000 cubic feet of fresh air per hour can be easily furnished for each patient. There are four inlet registers in each of the larger pavilion wards.

*Ventilation.*—The extraction system is employed in the simplest form, by providing chiefly for a natural upward outflow of the escaping foul air. In each of the large wards two ventilating openings with registers are placed in the chimney close to the ceiling—one over each fire-place. The clear outlet area, per bed, equals the inlet area, and is 72 square inches. The chimney is occupied mainly by a vent shaft. The smoke flue of each fire-place is a round earthen pipe worked into the corners of the chimney. The clear area of the shaft is 32 by 36 inches, and steam pipes are placed in it above the ventilating openings. The space under the domed roof of the building is ventilated by a small opening into the chimney to secure some change of air; and the roof space is also supplied with a small amount of air through small flues rising in the walls of the building from the basement. Ventilating flues nine inches in diameter are placed under the floor of the ward, each leading to the base of the chimney from a register under the foot of each bed. For each of the single rooms in the pavilions there is a vent-flue, with top and bottom registers alongside the chimney, leading to a warmed vent-chamber in the roof. There is a like arrangement for all rooms in the administration building, where a capacious vent-chamber under the roof has its outlet in the top of the tower over the main stairway. The special provisions for independent ventilation of all apartments, for water-closets, kitchens, and service rooms, have been noted.

The plumbing and fittings of water-closets, kitchen sinks, and the like, are all left open to view, or carried in accessible pipe-shafts. All soil-pipes have foot-ventilation and extend upwards to the outer air, and they also lead through traps to the sewer system. The water-closets are of the "Hopper" form, holding a large amount of water in the bowl, and simple in construction, working upon the syphon principle.

## Figures and Tables

**Figure f1:**